# Recognition of mulberry leaf diseases based on multi-scale residual network fusion SENet

**DOI:** 10.1371/journal.pone.0298700

**Published:** 2024-02-23

**Authors:** Chunming Wen, Wangwang He, Wanling Wu, Xiang Liang, Jie Yang, Hongliang Nong, Zimian Lan

**Affiliations:** 1 Key Laboratory of Intelligent Unmanned System and Intelligent Equipment, Education Department of Guangxi Zhuang Autonomous Region, Nanning, China; 2 Guangxi Key Laboratory of Hybrid Computation and IC Design Analysis, Nanning, China; 3 College of Electronic Information, Guangxi Minzu University, Nanning, China; 4 State Key Laboratory for Conservation and Utilization of Subtropical Agro-Bioresources, Guangxi University, Nanning, China; 5 Guangxi Sericulture Technology Promotion Center, Nanning, China; 6 Guangxi Agricultural Machinery Research Institute Company Limited, Nanning, China; 7 Key Laboratory of AI and Information Processing (Hechi University), Education Department of Guangxi Zhuang Autonomous Region, Hechi, China; Newcastle University, UNITED KINGDOM

## Abstract

Silkworms are insects with important economic value, and mulberry leaves are the food of silkworms. The quality and quantity of mulberry leaves have a direct impact on cocooning. Mulberry leaves are often infected with various diseases during the growth process. Because of the subjectivity and time-consuming problems in artificial identification of mulberry leaf diseases. In this work, a multi-scale residual network fusion Squeeze-and-Excitation Networks (SENet) is proposed for mulberry leaf disease recognition. The mulberry leaf disease dataset was expanded by performing operations such as brightness enhancement, contrast enhancement, level flipping and adding Gaussian noise. Multi-scale convolution was used instead of the traditional single-scale convolution, allowing the network to be widened to obtain more feature information and avoiding the overfitting phenomenon caused by the network piling up too deep. SENet was introduced into the residual network to enhance the extraction of key feature information of the model, thus improving the recognition accuracy of the model. The experimental results showed that the method proposed in this paper can effectively improve the recognition performance of the model. The recognition accuracy reached 98.72%. The recall and F1 score were 98.73% and 98.72% respectively. Compared with some other models, this model has better recognition effect and can provide technical reference for intelligent mulberry leaf disease detection.

## 1. Introduction

Sericulture industry is a combination of planting and breeding [1]. China is one of the oldest cocoon producers in the world. Mulberry leaves are food for silkworms and an important economic component in sericulture [2]. The quality and quantity of mulberry leaves have a direct impact on cocoon formation [3]. Mulberry trees are often infected with various diseases during growth, which induce poor leaf growth and cause a decrease in leaf yield and quality, which in turn adversely affects silkworm breeding. Among mulberry leaf diseases, red rust, powdery mildew, and brown spot are the most common [4]. Rapid and accurate identification of mulberry leaf diseases and timely adoption of control measures can help increase mulberry leaf production and income. At present, mulberry leaf diseases mainly rely on mulberry farmers or experts for on-site identification. Since manual identification is somewhat subjective, which is not only time-consuming and laborious, but also causes inaccurate identification leading to medication errors, it is important to realize efficient and intelligent mulberry leaf disease identification.

With the development of computer technology, significant progress has been made in crop disease recognition based on machine learning. Zhang Yan et al. [5] proposed an SVM based online recognition method for early blight in greenhouse tomatoes. This method is based on support vector machines and combines color and texture features to recognize and locate early blight spots in complex backgrounds. Ni Hongjun et al. [6] improved the basic RepVGG model and proposed a new classification model, RepVGG_ECA, which successfully classified five typical pests and diseases and healthy rice in six categories with a classification accuracy of 97.06%. It provides a valuable reference for identifying typical rice pests and diseases. Javidan et al. [7] used a new image processing algorithm and multi class support vector machines (SVM) to diagnose and classify grape leaf diseases, namely black measles, black rot, and leaf blight. Automatically separate disease symptom regions from healthy parts of leaves using K-means clustering. Finally, a classification accuracy of 98.97% was achieved. The above methods usually require feature extractors to extract the features of disease images, and the design of feature extractors depends on experience and has a certain degree of subjectivity. In addition, artificially constructed image features may not accurately reflect the disease characteristics of crops, which can affect recognition performance in practical use.

In recent years, along with the rapid development of machine learning, feature extraction based on deep learning has become a research hotspot. This class of methods does not rely on manual design and has received wide attention in the field of crop disease identification. Di Jie et al. [8] proposed a deep learning-based target detection model DF-Tiny-YOLO for apple leaf vein diseases to achieve faster and more effective automatic detection of apple leaf vein diseases, in view of the complexity and diversity of apple leaf veins as well as the difficulty in judging similar diseases. Ding Jie et al. [9] proposed a new apple leaf disease recognition model, RFCA ResNet, which has a dual attention mechanism and multi-scale feature extraction capability Experimental results show that RFCA ResNet significantly outperforms the standard CNN network model, with improved accuracy, precision, recall and F1 score of 89.61%, 56.66%, 72.76% and 58.77%. The method outperforms other methods, has better generalization, and has some theoretical relevance and practical value. Geetharamani et al. [10] proposed a deep convolutional neural network (Deep-CNN) based model for plant leaf disease recognition, which was trained using different training times and batch sizes, and after extensive experiments, the model achieved 96.46% recognition accuracy. Haque et al. [11] used Inception-v3 network framework and baseline training method to recognize maize disease images, and the overall recognition accuracy was 95.99% on the test dataset. Xie Shengqiao et al. [12] used migration learning and data enhancement techniques to achieve accurate classification of grape leaf disease images with an average recognition accuracy of 97.87% in order to achieve grape disease image recognition with small sample data. Pan Renyong et al. [13] proposed a DTS-ResNet-based method for apple leaf disease recognition with 98.73% recognition accuracy for the problems of low recognition accuracy and slow convergence of traditional convolutional neural networks in apple leaf disease recognition.

The most common challenge in using deep learning techniques for problem solving is that the training of parameters in the model requires massive amounts of data to support it. However, these data are often unlabeled and cannot be trained for machine learning models. Manual data calibration is too time-consuming. With migration learning, the relationships obtained for one type of data in a model training task can be easily applied to different problems in the same domain. Zhao Yue et al. [14] developed a Faster R-CNN network model based on TensorFlow with potato leaves as the research sample and used COCO initial weights for migration learning, which outperformed other network models and provided technical support for potato disease detection. Krishnamoorthy et al. [15] combined Inception-ResNetv2, a pre-trained deep convolutional neural network, with a transfer learning approach for the identification of rice leaf diseases, the parameters of the model were optimized for the identification task, and finally a recognition accuracy of 95.67% was obtained. Ahmed et al. [16] proposed two deep learning methods for palm leaf disease classification:Residual Network (ResNet) and Migration Learning with Inception ResNet. Both methods deal with variations in brightness and background, different scales of images, and inter-class similarity. The proposed model outperforms many recent studies in the field on the original and enhanced datasets, achieving 99.62% and 100% accuracy, respectively. Chen et al. [17] proposed a novel convolutional neural network for rice disease identification. The network combines the VGGNet architecture with the Inception module and incorporates a migration learning strategy in the training process. The results showed that the proposed model was able to achieve an average recognition accuracy of 92%. Xu et al. [18] proposed a multiscale convolutional global pooling neural network, which enhanced the AlexNet feature extraction by adding a convolutional layer and a new Inception module to the AlexNet model; to avoid too many parameters, a global pooling layer was used instead of the original fully connected layer and combined with migration learning to eliminate the model overfitting problem. The experimental results showed that the improved model has higher recognition accuracy. Nahiduzzaman et al. [19] proposed an explanation generation (XAI) framework combined with a novel lightweight PDS-CNN model for classifying disease-free leaves, leaf rust and leaf spot diseases from a newly established mulberry leaf image database. This XAI-based PDS-CNN model obtained a three-class classification accuracy of 95.05% with 0.53M parameters, 8 layers and 6.3MB size. Hu et al. [20] extracted the color features and texture features of tea diseases for disease spot segmentation, and finally completed the classification using VGG-16 network with an average recognition accuracy of 90%.

The above study shows that deep learning is being applied to crop disease identification. Deep learning models have higher performance compared to machine learning models and do not require manual feature extraction. However, most models learn by convolutional neural networks to obtain a single type of features, which cannot better characterize crop disease information and affect the recognition effect. In order to improve the recognition accuracy of the network model, this paper proposes a multi-scale network model incorporating attention mechanism. The contributions are as follows:

1. The network structure is improved, and the multi-scale is applied to solve the problem that the residual network extracts relatively single feature information of mulberry leaf diseases, which cannot express the main features of the image well.
2. In order to reduce the influence of non-diseased areas on model recognition, Squeeze-and-Excitation Networks (SENet) is introduced, which makes the focus of the model basically dominated by diseased areas and effectively improves the recognition accuracy of the network.
3. To address the problem that the output of the ReLU activation function may be 0, which will result in the gradient not being updated. The ELU activation function is used to replace the ReLU activation function.

## 2. Materials and methods

### 2.1 Convolutional Neural Networks

Convolutional Neural Networks (CNN) is a multi-processing layer network model, which consists of convolutional layer, pooling layer and fully connected layer, etc. Through convolutional operations, the Convolution layer can extract local features of the image, and then a new layer of feature map can be obtained after the activation function [21]. The computational formula of the convolution layer is expressed as:

$$\begin{array}{c} x_{j}^{l}=f(\sum_{i\in M_{j}}x_{i}^{l-1}k_{ij}^{l}+b_{j}^{l}) \end{array}$$ (1)

where $x_{j}^{l}$ is the output of *j* neuron in layer *l*; $x_{i}^{l-1}$ is the output of *i* neurons in layer *l*; *l* is the layer number; *M*_*j*_ is the set of input features.

The nonlinear activation function uses the Rectified Linear Units (ReLU) function, i.e.

$$\begin{array}{c} f\left(x\right)=\{\begin{array}{cc} 0 & \left(x<0\right)\\ x & \left(x>0\right) \end{array} \end{array}$$ (2)

where *f*(*x*) is the ReLU function; *x* is the ReLU function independent variable.

The pooling layer can reduce the dimensionality of the feature map to reduce the probability of overfitting. The pooling formula is expressed as:

$$\begin{array}{c} x_{j}^{l}=f_{down}\left(x_{i}^{l-1}\right) \end{array}$$ (3)

where *f*_*down*_(⋅) is the downsampling function.

The fully connected layer is located after the pooling layer, which further dimensionalizes the features and feeds them into the SoftMax classifier. CNN is trained using the gradient descent method to minimize the loss function. The loss function calculation formula is expressed as:

$$\begin{array}{c} L(W,b)=-\sum_{i=1}^{N}\sum_{j=1}^{C}I\{{\hat{y}}_{\text{i}}=j\}\text{lg}\;p_{i}^{j} \end{array}$$ (4)

where *W* is the weight; *b* is the bias term; ${\hat{y}}_{\text{i}}$ is the first training sample expectation;*N* is the total number of training samples; C is the number of training sample categories; *I* is the indicator function; *j* is the training sample category; $p_{i}^{j}$ is the *i* training sample *j* category prediction probability.

### 2.2 Residual network

When the network model is deeper in layers, the gradient may disappear or explode, which leads to the network not converging. The gradient disappearance will lead to the model weights not being updated, which will cause the model to fail to learn. Gradient explosion can cause the model weights to update too much, which can cause the model to be unstable and unable to learn effectively. To solve this problem, He et al. [22] proposed Deep Residual Networks (ResNet) to simplify the training of deeper networks. The core of the residual network lies in the ResNet residual block structure, where the residual network bypasses the inputs of the later layers to solve the gradient disappearance or gradient explosion.

The residual network still lets the nonlinear layer satisfy *H*(*x*, *w*_*h*_) and then introduces a short connection from the input directly to the output of the nonlinear layer. The mapping has the following equation:

$$\begin{array}{c} y=H(x,w_{h})+x \end{array}$$ (5)

where *y* denotes the residual output of the neural network and *H*(*x*, *w*_*h*_) is the residual value of the input convolutional layer. *x* denotes the input to the residual unit during training.

**Fig 1:**
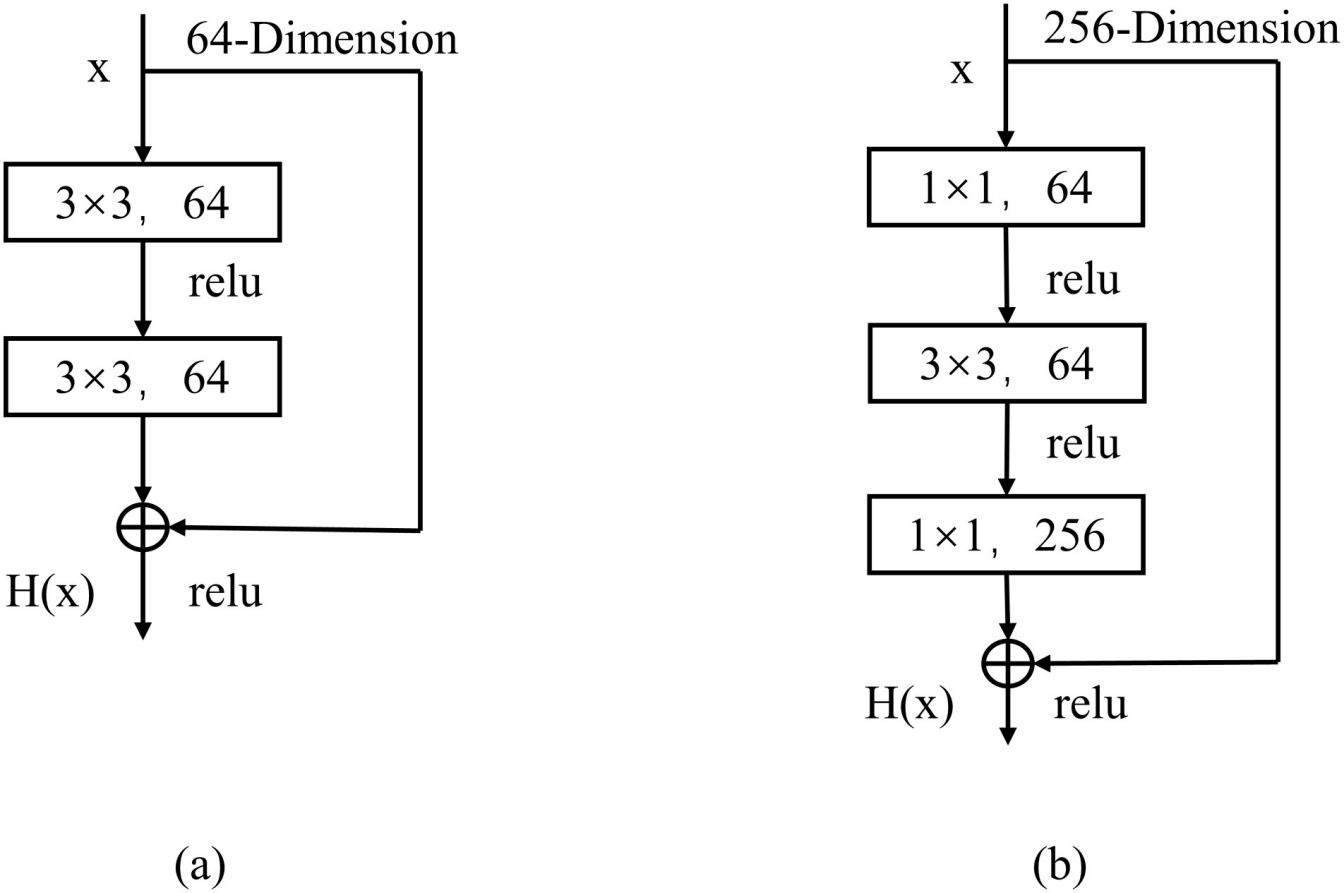
Residuals module. (a) building block; (b) bottleneck block.

For neural networks of different depths, two types of residual blocks are used, namely building block and bottleneck block, and the specific structures of these two residual blocks are shown in Fig 1.

The conventional residual module, which consists of two 3 × 3 convolutional layers stacked on top of each other, is not very effective in practice as the depth of the network increases.

The bottleneck residual module is composed of three convolutional layers, 1 × 1, 3 × 3, and 1 × 1. The 1 × 1 convolutional can up-dimension or down-dimension the number of channels, so that the 3 × 3 convolutional can perform the convolutional operation with relatively low-dimensional input and improve the computational efficiency.

Due to the degradation of the network caused by too many layers of network too deep, the residual neural uses the method of bypassing the input of the later layers to solve this problem, but there are some shortcomings.

1. When the model is built, as the depth of the network increases, the number of samples required for training should also increase so as to maximize the performance of the network and thus obtain better recognition results. However, due to the limited sample size of the dataset, if the network depth is blindly increased, it may cause the phenomenon of overfitting.
2. In the residual neural network, all the convolutional layers use a convolutional kernel of 3 × 3 size for convolutional operation, which has some limitations on the extraction of feature information in the image, and the extracted feature information is relatively single and cannot express the main features of the image well.

### 2.3 Multiscale residual convolution module

To address the shortcomings of the residual network, this paper constructs the residual network by using multi-scale convolutional kernels of different sizes instead of single convolutional kernels to enrich the feature extraction information. The 3 × 3 single-scale convolutional kernels connected in series in the backbone part are replaced with 1 × 1, 3 × 3 and 5 × 5 sized convolutional kernels connected in parallel. In convolutional neural networks, the selection of convolutional kernels is very critical. Large-sized convolutional kernels can obtain a large amount of information and focus more on the extraction of the overall information; small-sized convolutional kernels can obtain more detailed information and focus on the extraction of local subtle information.

**Fig 2:**
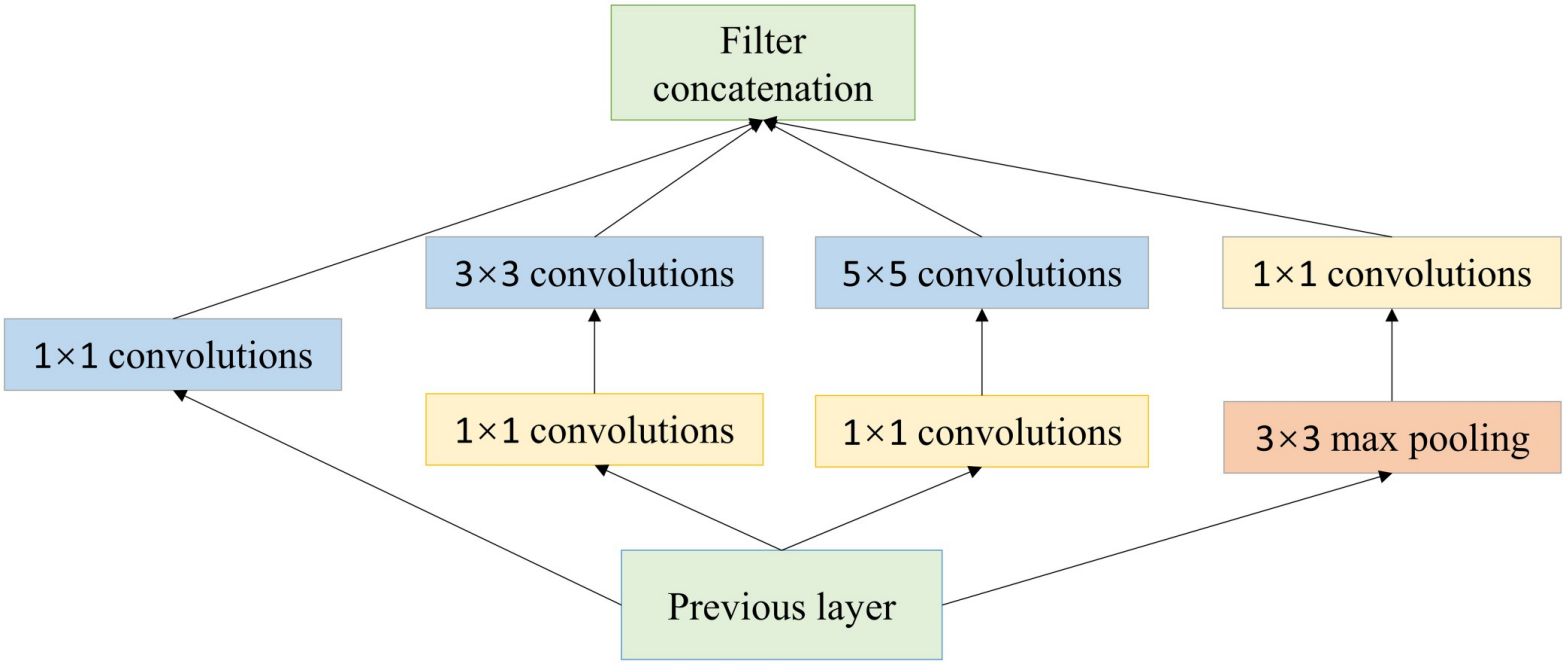
Inception module.

The use of different sizes of convolutional kernels to extract image feature information, if only the convolutional kernels are simply stacked, this makes the depth of the network increase, which makes the computational complexity increase, resulting in overfitting phenomenon. Therefore, this paper adopts the structure of Inception module, using different sizes of convolutional kernels to extract features on the same layer to expand the width of the network and avoid the overfitting phenomenon caused by the network being too deep. Inception module is shown in Fig 2. The Inception module adopts a parallel structure of convolutional layers and passes the feature maps to each of the four branches. The main purpose of the 1 × 1 convolutional layer in the yellow part is to reduce the dimensionality of the features, reduce the number of parameters, and thus reduce the computational effort of the network.

The improved residual module is a multi-scale convolutional parallel structure that replaces the backbone part of the conventional residual module with an Inception module. The 3 × 3 single-scale convolutional kernels connected in series in the backbone part are replaced with 1 × 1, 3 × 3 and 5 × 5 size convolutional kernels connected in parallel. Using multi-scale convolutional kernels for perception at different scales. By setting different step sizes and fill sizes in the convolutional layers, they have the same feature dimension after convolutional operations. Facilitate the fusion of features and enrich the feature information of the feature map.

In the Inception module, the fusion weights for each convolution are not explicitly set. Instead, the fusion of features is achieved by feature concatenation operations, not by weighted summation of weights. Each branch has its own convolution operation in the Inception module, such as a 1 × 1, 3 × 3, 5 × 5 convolution kernel or pooling operation. The feature maps extracted by these branches are spliced in the channel dimension to form a deeper feature representation. With feature splicing, the feature information extracted from different branches is seamlessly fused together without introducing additional weighting parameters. This approach allows for a more concise model and makes full use of feature information of different sizes and abstraction levels.

### 2.4 Squeeze-and-Excitation Networks

The Squeeze-and-Excitation Networks(SENet) is a lightweight channel attention module with a simple structure that makes it easy to apply to network models to improve their performance [23]. The main purpose is to learn the correlation between channels to get the channel-specific attention, so that the model can focus more on the important features of the image and reduce the influence of complex background factors to improve the model training effect. The difference with traditional CNN is that SENet recalibrates the previously obtained features by two operations, Squeeze and Excitation. The first is the Squeeze operation, which performs feature compression along the spatial dimensions, turning each two-dimensional feature channel into a real number, so that layers close to the input also get a global view of the sensation. The algorithm formula is expressed as:

$$\begin{array}{c} F_{sq}\left(u_{c}\right)=\frac{1}{H\times W}\sum_{i=1}^{H}\sum_{j=1}^{W}u_{c}\left(i,j\right) \end{array}$$ (6)

where **u**_*c*_ is the *c*th feature map with input size H × W.

Next is the Excitation operation, which mainly consists of two fully connected layers and two activation functions, and the algorithmic formula is expressed as:

$$\begin{array}{c} s=F_{ex}\left(F_{sq}\left(u_{c}\right),W\right)=\sigma \left(W_{2}\delta \left(W_{1}F_{sq}\left(u_{c}\right)\right)\right) \end{array}$$ (7)

where: *σ* is the ReLU activation function; *δ* is the Sigmoid activation function; W_1_ is the first fully connected layer; W_2_ is the second fully connected layer; **F**_*sq*_(**u**_*c*_) is the output value after Excitation operation.

**Fig 3:**
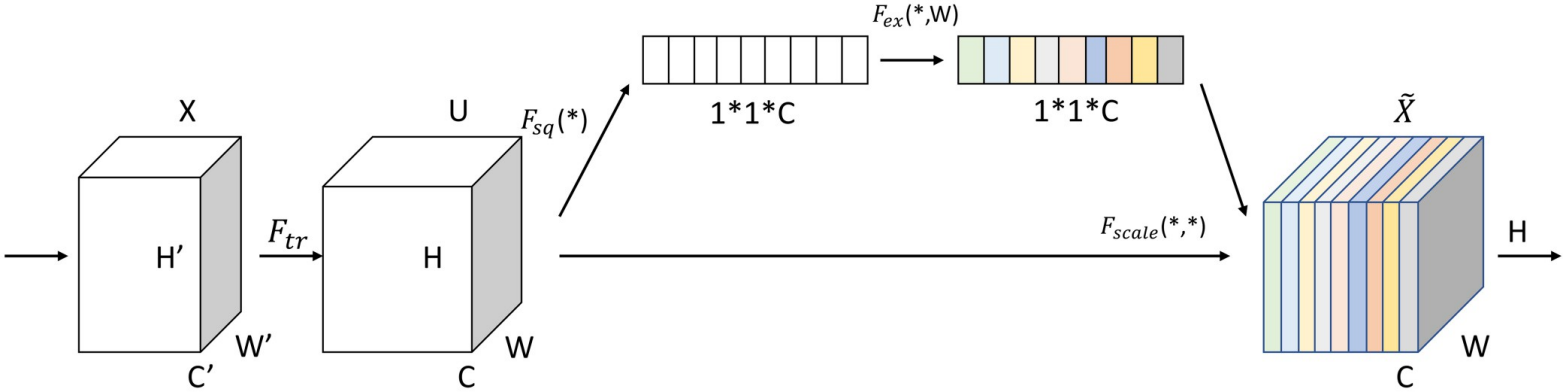
SE module structure.

The SE module consists of 3 main parts, compression, excitation and reweighting, as shown in Fig 3.

Given an input feature map X, let it go through the *F*_*tr*_ operation to generate a feature map U [24].

1. Compression part:
  The input feature image is compressed by global average pooling, which compresses the two-dimensional features (H*W) of each channel into 1 real number, transforming the feature map from [H,W,C] to [1,1,C]. The essence of the compressed feature is a vector with no spatial dimension, only channel dimension.
2. Stimulus component:
  Weights are generated for each channel that can be considered as its importance, and the number of output weight values is the same as the number of channels in the input feature map.
3. Re-weighting part:
  After the input feature image is compressed and excited, the original input features are redefined by multiplying the channel-by-channel weighting onto the corresponding channel of the original input feature map. In this paper, the SE attention module is added to the basic blocks of ResNet. Specifically, after each convolutional layer, the SE attention module is applied to the output of the convolutional layer. Attention weights can be applied by multiplying the output of the convolutional layer with the output of the SE attention module. The implementation is mainly to obtain the information of each channel of the input features through global average pooling, and subsequently apply two fully connected layers in order to realize the compression and excitation operations in the module, so that the reweighting of the output of each residual module can be realized, which makes the network pay more attention to the important feature information in the feature image and thus enhances the recognition effect of the model.

### 2.5 Transfer learning

Transfer learning is done by simply adapting a pre-trained model and applying it to a new task [25]. The trained convolutional layer can perform image feature extraction, and the extracted feature vectors can be input to the fully connected layer to obtain good classification recognition performance, so the feature vectors extracted by the convolutional layer can be used as the streamlined vectors of images. Therefore, the pre-trained convolutional layer and the newly designed fully-connected layer form a new neural network model, and the model can be trained with small sample data to obtain better performance.

**Fig 4:**
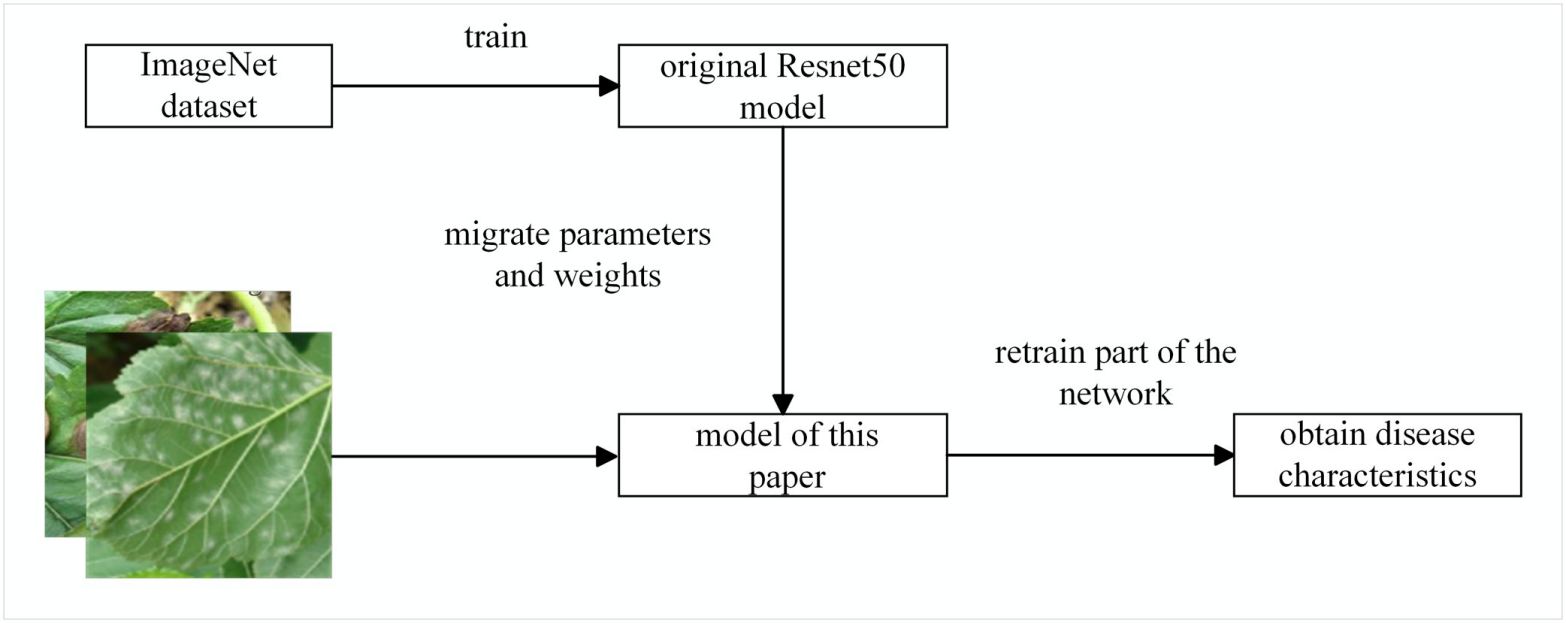
Transfer learning process.

As shown in Fig 4, this paper applies the image classification knowledge learned on the large dataset imageNet to mulberry leaf disease recognition through migration learning. Constructing the model of this paper based on the network model obtained after migration learning is faster than constructing and training a new network with random initialization.

### 2.6 Activation function replacement

The activation function is an important part of the convolutional neural network that introduces nonlinear factors to the neural network, thus allowing the neural network to fit a variety of nonlinear models and enhance the expressive power of the model. The activation function used by ResNet is the Rectified Linear Unit (ReLU), which is expressed as:

$$\begin{array}{c} ReLU\left(x\right)=\left\{ \begin{array}{c} x\;,x>0\\ 0\;,x⩽0 \end{array}\right. \end{array}$$ (8)

The ReLU activation function is essentially a segmented linear function. It leaves all positive values unchanged while the nonpositive values become 0. This is known as unilateral inhibition. Since the output of the ReLU activation function may be 0, this can lead to the gradient not being updated, which in turn leads to the dying ReLU problem.

Unlike the ReLU activation function, the Exponential Linear Unit (ELU) is expressed as:

$$\begin{array}{c} ELU\left(x\right)=\{\begin{array}{c} x,x⩾0\\ e^{x}-1\;,x<0 \end{array} \end{array}$$ (9)

**Fig 5:**
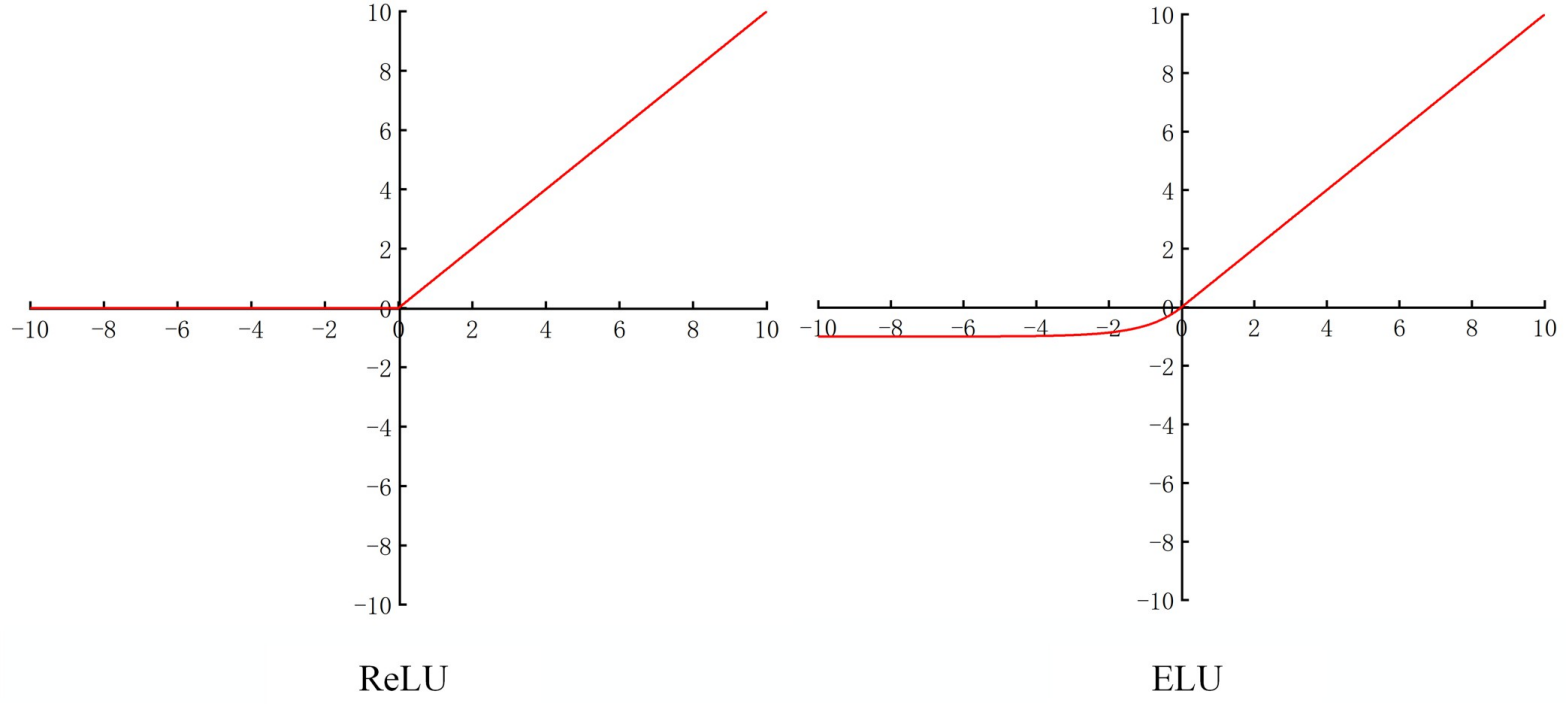
ReLU activation function and ELU activation function.

The ELU activation function has the same expression for positive intervals as ReLU, which also avoids the gradient vanishing problem. At the same time its gradient is not zero for negative intervals, thus avoiding the dying ReLU problem, thus allowing the network to learn more features. Fig 5 shows the graphs of the two activation functions.

### 2.7 Total network architecture and process

**Fig 6:**
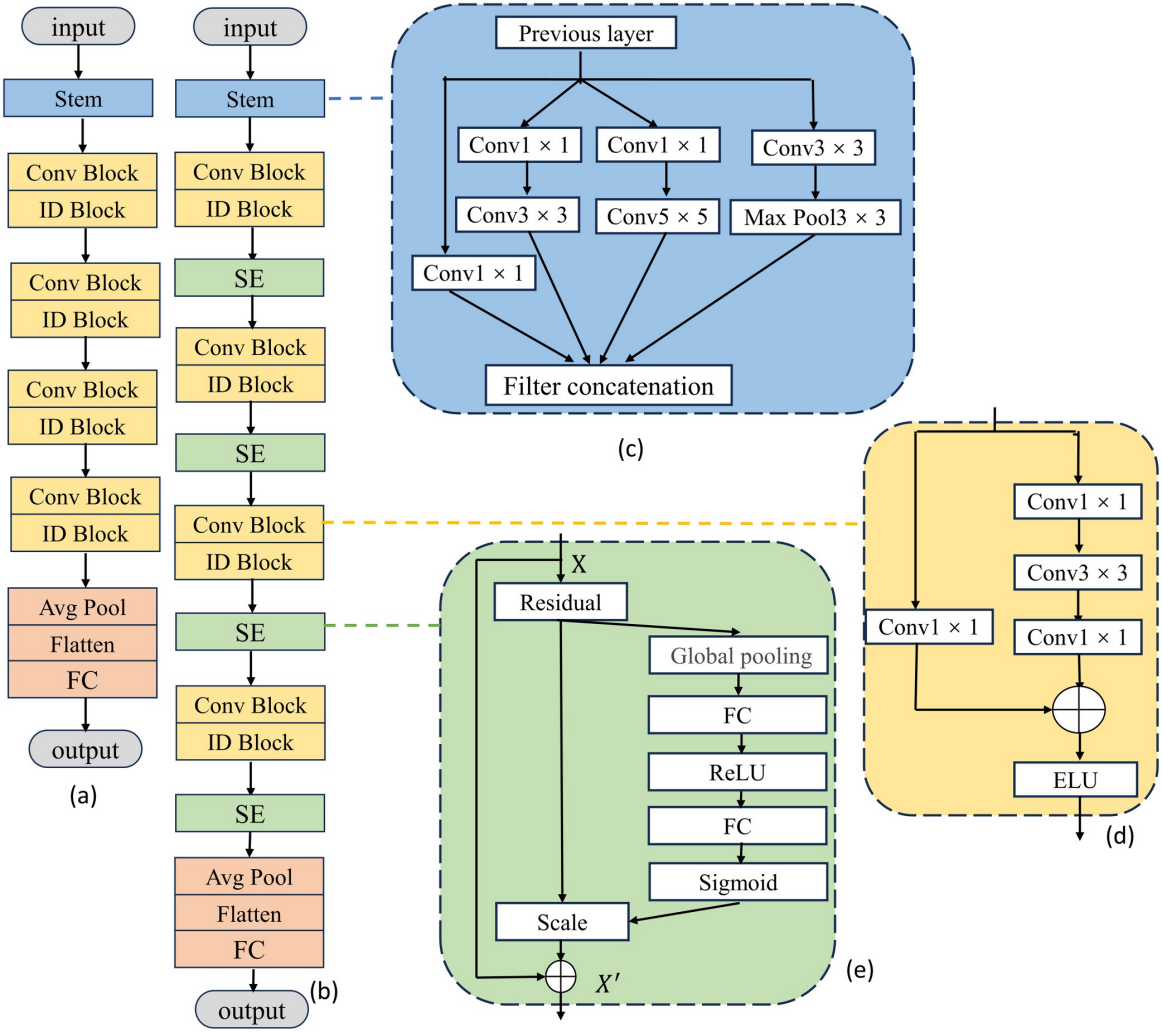
Total network architecture.

In this paper, a multiscale residual network fused with a squeezed excitation network is proposed for mulberry leaf disease recognition. The total network architecture is shown in Fig 6. In the figure, (a) shows the original resnet50 model and (b) shows the improved resnet50 model. (c) shows the improvement made to the 7 × 7 convolution of the first layer of the resnet50 model. Multiple convolution kernels of different scales of 1×1, 3×3, and 5×5 are used to replace the 7×7 convolution kernels in the base network. The output feature maps of the 1×1, 3×3, and 5×5 convolutional layers are spliced together to enrich the final feature expressiveness. (d) in the figure shows the residual block in resnet50, in which the ReLU activation function is replaced by the ELU activation function to avoid the dying ReLU problem. (e) shows the structure of SENet combined with resnet50. After each convolutional layer, the SE attention module is applied to the output of the convolutional layer.

**Fig 7:**
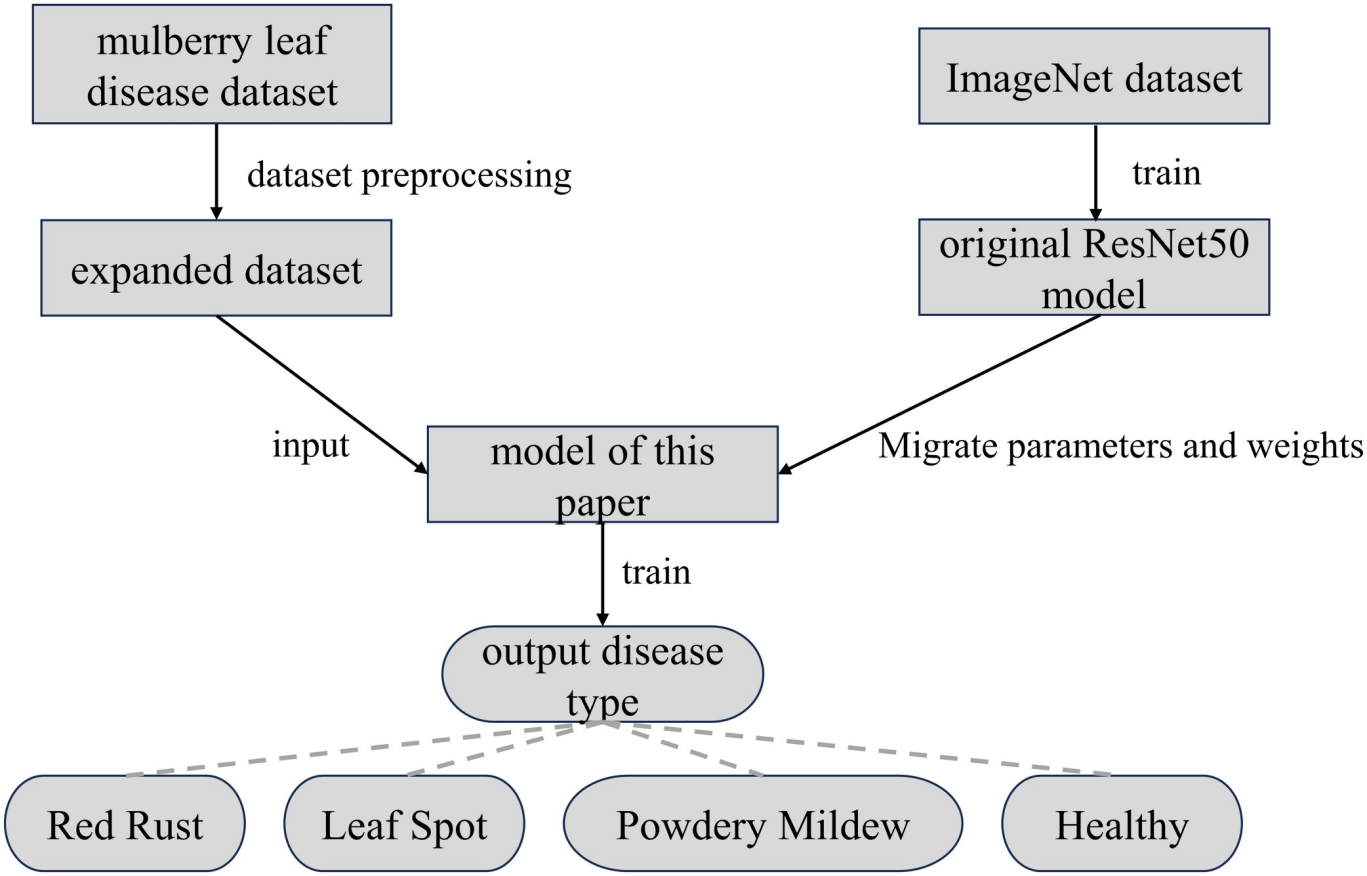
Network process.

The process of running the model in this paper is introduced as shown in Fig 7. The dataset is preprocessed and the expanded dataset is imported into the model. At the same time, the parameters and weights of the original resnet50 model trained on ImageNet dataset are migrated to the model of this paper. Then the training of this paper’s model is started to output the diseases corresponding to mulberry leaves.

## 3. Results and discussion

### 3.1 Dataset source

**Fig 8:**
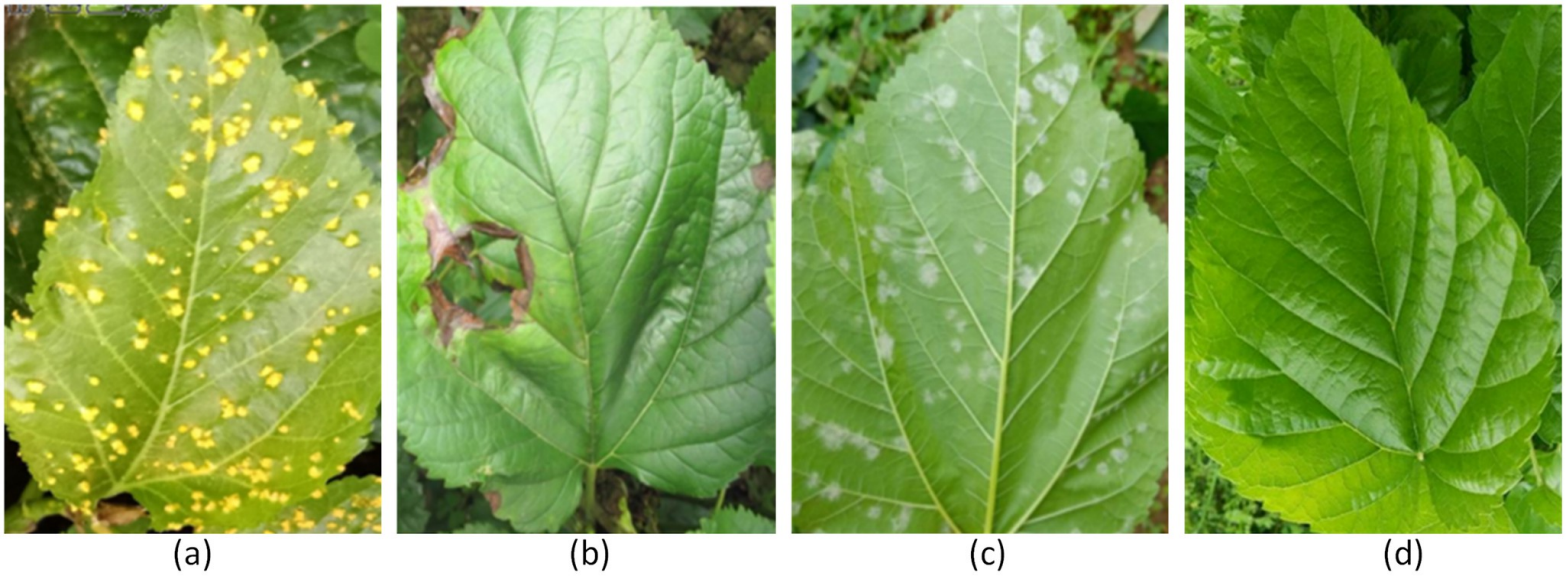
Mulberry leaf disease dataset category. (a) Red Rust; (b) Leaf Spot; (c) Powdery Mildew; (d) Healthy.

The dataset used in this paper is primarily from the kaggle dataset platform. The dataset is publicly available. A few images were taken by us. These images were taken at the Sericulture Technology Promotion Station of Guangxi Zhuang Autonomous Region, Nanning, Guangxi Zhuang Autonomous Region, China. We obtained permission from the Sericulture Technology Promotion Station of Guangxi Zhuang Autonomous Region for collecting the images. The images of mulberry leaf diseases are shown in Fig 8.

1. The typical symptom of Red Rust is that mulberry leaves are infested with scattered round shiny dots, fat and elevated, and the color changes from yellow to orange, and the epidermis is ruptured and covered with orange-yellow powder, i.e. rust spores.
2. The typical symptom of Leaf Spot is that mulberry leaves first appear gray-brown dot-like spots, expanding into round or irregular-shaped spots with concentric whorls, and the spots lose water and dry out.
3. The typical symptoms of Powdery Mildew are white scattered mildew spots on the back of the leaves, which gradually expand and can cover the entire leaf back. The surface of the mildew is powdery, that is, the mycelium and conidia of the pathogenic fungus.

### 3.2 Data pre-processing

The mulberry leaf disease dataset contains three mulberry leaf disease images and one healthy mulberry leaf image. In order to balance the images in each category, the dataset was expanded for certain categories by brightness enhancement, contrast enhancement, level flipping and adding Gaussian noise. As shown in Table 1, the proportion of training set, validation set and test set is 8:1:1.

**Table 1:** Dataset division table.

Type	Training Set	Validation Set	Test Set
Healthy	581	73	73
Red Rust	548	68	68
Leaf Spot	541	67	67
Powdery Mildew	460	57	57

### 3.3 Experimental platform and experimental parameter settings

The configuration of the experimental environment in this study is shown in Table 2, with the learning rate set to 0.001 and the number of iterations set to 100, where the batch size of the training set at each iteration is set to 32. In the training process of the network, the input image size is uniformly 256 pixels × 256 pixels. In order to avoid the feature map becoming smaller and smaller after convolution and the loss of image edge information, the padding operation is used to fill the pixels along the edges of the image during convolution, which is generally done with 0. The filling process of edge information can extract edge information more fully and avoid edge information loss.

**Table 2:** Experimental environment configuration.

Experimental environment	Configuration parameters
Operating system	Windows10
GPU	NVIDIA GeForce RTX 3090
Deep learning framework	Pytorch 1.11
Programming language	Python3.8
GPU acceleration libraries	CUDA 11.3 CUDNN 8

### 3.4 Evaluation indicators

In this experiment, the accuracy rate A, precision rate P, recall rate R and F1 score are used as evaluation criteria. The corresponding formulas for their evaluation metrics are shown below.

$$\begin{array}{c} A=\frac{TP+TN}{TP+TN+FN} \end{array}$$ (10)

$$\begin{array}{c} P=\frac{TP}{TP+FP} \end{array}$$ (11)

$$\begin{array}{c} R=\frac{TP}{TP+FN} \end{array}$$ (12)

$$\begin{array}{c} F1=2\times \frac{P\times R}{P+R} \end{array}$$ (13)

TP is the number of positive samples predicted to be positive; TN is the number of negative samples predicted to be negative; FP refers to the number of positive samples predicted to be negative; and FN refers to the number of negative samples predicted to be positive.

Accuracy A is defined as the percentage of correctly predicted outcomes to the total sample. In the case of unbalanced samples, the high accuracy rate obtained is not very meaningful, and other indicators are needed to evaluate the merits of the model.

Accuracy P is for the predicted outcome and is defined as the probability of actually being a positive sample among all samples that are predicted to be positive.

Recall R is for the original sample and is defined as the probability of a positive sample being predicted out of the actual positive samples. The F1 score considers both the precision rate and the recall rate, so that both are maximized at the same time and a balance is achieved.

### 3.5 Extraction of key features of disease spots

The heat map obtained by Grad-CAM can analyze the areas of interest of the network for a certain category, and use the areas of interest of the network to in turn analyze whether the network learns the correct features or information.

Heat maps are usually used to classify images into categories, similar to infrared imaging maps, where high temperature areas appear red and low temperature parts appear blue. Similarly, the heat map is used to show how much attention the neural network pays to each part of the image in the form of weights. In the heat map, the red part is the part with more attention and the blue part is the part with less attention.

**Fig 9:**
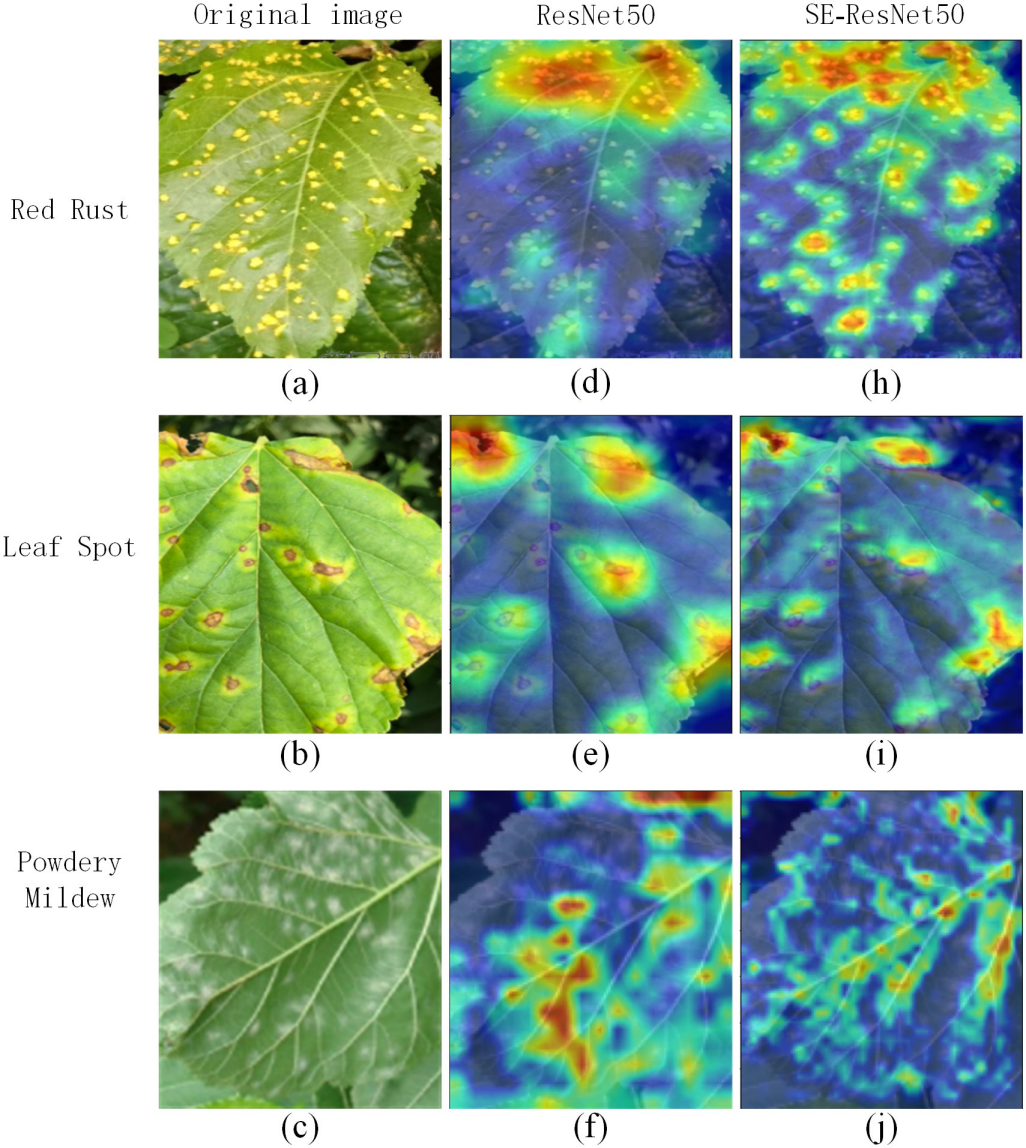
Heat maps of different diseases. (a-c) are the original images of the three diseases. (d-f) are the thermal maps under the ResNet model. (h-j) are the thermal maps under the ResNet model after adding the SE module.

According to Fig 9, it can be seen that when targeting the same image, the model without adding the SE module does not pay enough attention to the fine points, and will consider some non-diseased areas as the focus of attention, which will have a certain impact on the recognition accuracy of the model. After adding the SE module, the focus of the model is basically on the diseased areas, and the influence of non-diseased areas is reduced, which can effectively improve the recognition accuracy of the network.

### 3.6 Ablation experiment

In this paper, the classification performance of the proposed method was verified by ablation experiments. The experiments are compared and validated against the training results of ResNet50. The results are shown in Table 3. In Table 3, IN denotes the use of the improved residual module, SE denotes the embedded attention mechanism module, and INSE-ResNet50 denotes the final model of this paper. As can be seen from Table 3, the ResNet50 model without any improvement was the minimum in all indicators. The accuracy was 93.75%, precision was 94.10%, recall was 93.74%, and F1 score was 93.60%. The model using the improved residual module while embedding the SE module had maximum values for all metrics. The accuracy was 98.72%, precision was 98.71%, recall was 98.73%, and F1 score was 98.72%. Embedding the SE module in the network and training the model using the improved residual module increased its accuracy by 1.92% and 2.75%, respectively. There was also some improvement in precision, recall, and F1 score. Networks trained with the improved residual module while embedding the SE module were able to increase the accuracy by 4.97% and achieved a higher F1 score. The results showed that the proposed method achieved better results in terms of accuracy and F1 score, and the overall performance was improved compared to that before the improvement.

**Table 3:** Ablation experiment.

Model	IN	SE	Accuracy	Precision	Recall	F1 Score
ResNet50	-	-	93.75%	94.10%	93.74%	93.60%
SE-ResNet50	-	✓	95.67%	96.05%	95.67%	95.70%
IN-ResNet50	✓	-	96.50%	96.39%	96.45%	96.40%
INSE-ResNet50	✓	✓	98.72%	98.71%	98.73%	98.72%

### 3.7 Loss and accuracy

**Fig 10:**
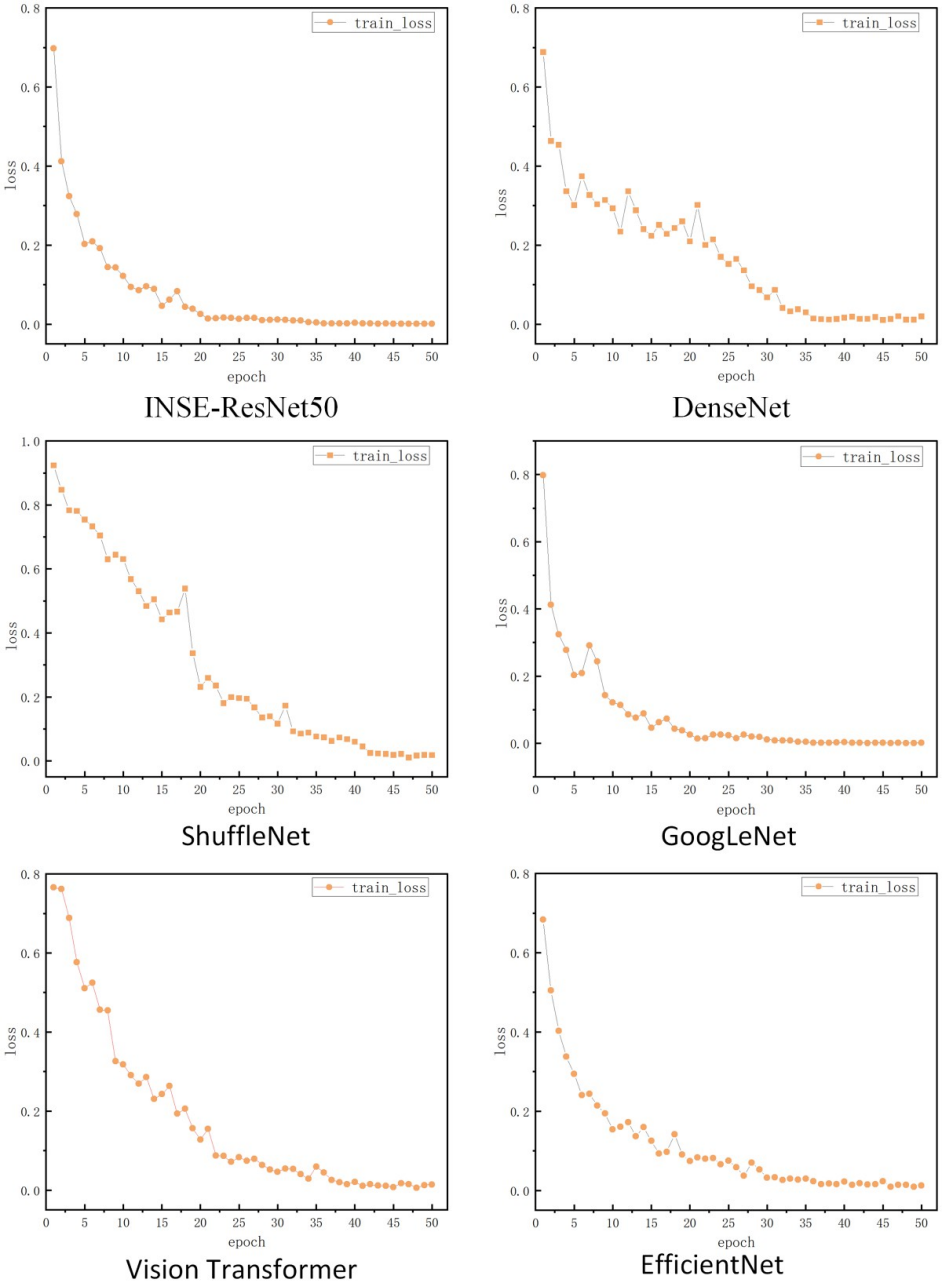
Images of loss rates for different models.

The loss rate curves of the four models are shown in Fig 10. INSE-ResNet50 was the improved model in this paper. The horizontal coordinate epoch indicated the number of model iterations, 50 iterations for each model respectively, and the vertical coordinate loss indicated the loss rate of the model. Overall the loss rate of all four models on the training set trended down. From the loss curves of each model on the training set, the loss curve of INSE-ResNet50 converged from the 21st iteration, the loss curve of DenseNet converged from the 36th iteration, the loss curve of ShuffleNet converged from the 42th iteration, the loss curve of GoogLeNet converged from the 35th iteration, the loss curve of Vision Transformer converged from the 37th iteration, and the loss curve of EfficientNet converged from the 30th iteration. It can be seen that the model in this paper converged faster and the model performance was better.

**Fig 11:**
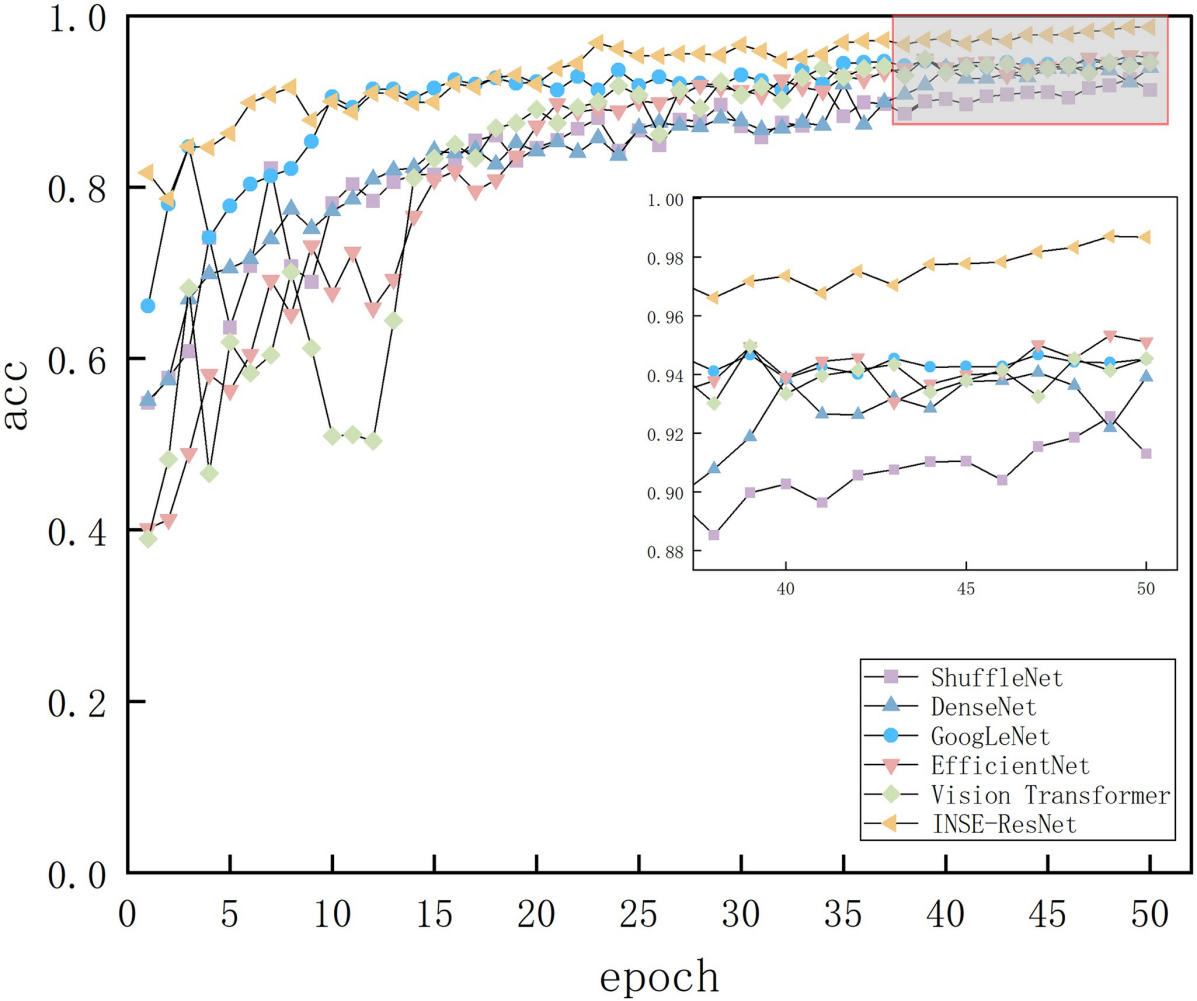
Accuracy images of different models.

The accuracy curves of the four models are shown in Fig 11. INSE-ResNet50 was the improved model in this paper. Overall the accuracy of the four models showed an increasing trend, and eventually the accuracy converged. The horizontal coordinate epoch indicates the number of model iterations, each model has 50 iterations, and the vertical coordinate acc indicates the accuracy of the model. The accuracy of INSE-ResNet50 was 98%, EfficientNet was 95%, Vision Transformer was 94%, GoogLeNet was 94%, DenseNet was 93%, and ShuffleNet was 91%. It can be seen that the model in this paper had the highest recognition accuracy and better classification than the other models. classification was better than other models.

## 4. Conclusion

In this paper, we proposed a mulberry leaf disease recognition model based on multiscale feature extraction and SE attention mechanism. To improve the network recognition accuracy, a new residual module was constructed using multiple convolutional kernels instead of a single convolutional kernel for feature extraction, and more feature information was obtained by network widening to avoid the overfitting phenomenon caused by too deep network stacking. After using the improved residual module, the accuracy, precision, recall, and F1 score of the model were improved by 2.75%, 2.29%, 2.71%, and 2.8%, respectively. Adding an attention mechanism to the residual module allows the attention of the neural network to focus on the focal channel. This reduces the influence of non-spot regions and improves the model performance. After adding the attention mechanism, the accuracy, precision, recall, and F1 score of the model were improved by 1.92%, 1.95%, 1.93%, and 2.1%, respectively. In addition to the ablation experiments, the model in this paper was compared with ShuffleNet, DenseNet, GoogLeNet, EfficientNet and Vision Transformer. The experimental results showed that the proposed method can effectively improve the recognition performance of the model with 98.72% recognition accuracy and better classification than other models, which provide a technical reference for intelligent mulberry leaf disease detection. Considering that the model needs to be deployed on mobile devices, factors such as computational efficiency and memory consumption also need to be considered in model improvement. Subsequent lightweighting of the model is needed to improve computational efficiency while maintaining high accuracy.
