# Correction: Recognition of mulberry leaf diseases based on multi-scale residual network fusion SENet

**DOI:** 10.1371/journal.pone.0301490

**Published:** 2024-03-26

**Authors:** Chunming Wen, Wangwang He, Wanling Wu, Xiang Liang, Jie Yang, Hongliang Nong, Zimian Lan

The published funding statement is incorrect. The correct funding statement is as follows: This work was supported by the Key Laboratory of AI and Information Processing (Hechi University), Education Department of Guangxi Zhuang Autonomous Region (No. 2022GXZDSY009), Innovation Project of Guangxi Graduate Education (No. YCSW2022XXXX), College Student Innovation and Entrepreneurship Training Program (No. S202210608188X, No. 201810608082) and the Natural Science Foundation of Guangxi Province (No. 2018GXNSFAA281164). The funders had no role in study design, data collection and analysis, decision to publish, or preparation of the manuscript.
